# Bevacizumab-Based Chemotherapy Combined with Regional Deep Capacitive Hyperthermia in Metastatic Cancer Patients: A Pilot Study

**DOI:** 10.3390/ijms18071458

**Published:** 2017-07-06

**Authors:** Girolamo Ranieri, Cristina Ferrari, Alessandra Di Palo, Ilaria Marech, Mariangela Porcelli, Gianmarco Falagario, Fabiana Ritrovato, Luigi Ramunni, Margherita Fanelli, Giuseppe Rubini, Cosmo Damiano Gadaleta

**Affiliations:** 1 Diagnostic and Interventional Radiology Unit with Integrated Section of Translational Medical Oncology, National Cancer Research Centre, IRCCS Istituto Tumori “Giovanni Paolo II”, Viale Orazio Flacco 65, 70124 Bari, Italy; ferrari_cristina@inwind.it (C.F.); dipaloalessandra@gmail.com (A.D.P.); ilariamare@tin.it (I.M.); marypor@libero.it (M.P.); gianmacu91@gmail.com (G.F.); f.ritrovato@live.it (F.R.); gigezio@libero.it (L.R.); cd.gadaleta@gmail.com (C.D.G.); 2 Nuclear Medicine Unit, D.I.M., University of Bari “Aldo Moro”, Piazza G. Cesare 11, 70124 Bari, Italy; margherita.fanelli@uniba.it (M.F.); giuseppe.rubini@uniba.it (G.R.)

**Keywords:** tumor angiogenesis, bevacizumab, hyperthermia, chemotherapy, tumor response, metastatic cancer

## Abstract

As an angiogenesis inhibitor, bevacizumab has been investigated in combination with different chemotherapeutic agents, achieving an established role for metastatic cancer treatment. However, potential synergic anti-angiogenic effects of hyperthermia have not tested to date in literature. The aim of our study was to analyze efficacy, safety, and survival of anti-angiogenic-based chemotherapy associated to regional deep capacitive hyperthermia (HT) in metastatic cancer patients. Twenty-three patients with metastatic colorectal (*n* = 16), ovarian (*n* = 5), and breast (*n* = 2) cancer were treated with HT in addition to a standard bevacizumab-based chemotherapy regimen. Treatment response assessment was performed, according to the modified Response Evaluation Criteria for Solid Tumors (mRECIST), at 80 days (timepoint-1) and at 160 days (timepoint-2) after therapy. Disease Response Rate (DRR), considered as the proportion of patients who had the best response rating (complete response (CR), partial response (PR), or stable disease (SD)), was assessed at timepoint-1 and timepoint-2. Chi-squared for linear trend test was performed to evaluated the association between response groups (R/NR) and the number of previous treatment (none, 1, 2, 3), number of chemotherapy cycles (<6, 6, 12, >12), number of hyperthermia sessions (<12, 12, 24, >24), and lines of chemotherapy (I, II). Survival curves were estimated by Kaplan-Meier method. DRR was 85.7% and 72.2% at timepoint-1 and timepoint-2, respectively. HT was well tolerated without additional adverse effects on chemotherapy-related toxicity. Chi-squared for linear trend test demonstrated that the percentage of responders grew in relation to the number of chemotherapy cycles (*p* = 0.015) and to number of HT sessions (*p* < 0.001) performed. Both overall survival (OS) and time to progression (TTP) were influenced by the number of chemotherapy cycles (*p* < 0.001) and HT sessions (*p* < 0.001) performed. Our preliminary data, that need to be confirmed in larger studies, suggest that the combined treatment of bevacizumab-based chemotherapy with HT has a favorable tumor response, is feasible and well tolerated, and offers a potentially promising option for metastatic cancer patients.

## 1. Introduction

Angiogenesis is a critical process for tumor growth and invasion. For this reason, the new treatment approach based on angiogenesis inhibitors is becoming a promising target in cancer therapy.

Bevacizumab, the first anti-angiogenic approved drug, was developed as a monoclonal antibody that binds and inactivates soluble Vascular Endothelial Growth Factor-A (VEGF-A), the isoform responsible for pathologic angiogenesis found to be up-regulated in various human tumors [1,2,3,4].

Clinically, bevacizumab has been investigated in combination with a range of chemotherapeutic agents for the treatment of different metastatic cancer; its efficacy was tested in metastatic colorectal cancer, in combination with irinotecan and 5-fluorouracil/leucovorin [5,6], and in non-small-cell lung cancer, in combination with paclitaxel and carboplatin [7,8]. In addition to metastatic lung and colorectal cancer, bevacizumab was recently also approved for the treatment of metastatic renal cell, ovarian, and breast cancer [9,10,11]. However, the outcome resulting from anti-angiogenic therapy, is variable [6,8,10,11,12,13]. While the approvals in lung and colorectal cancer were based on an improved overall survival (OS) with bevacizumab [6,8], the approvals in metastatic renal, ovarian, and breast cancer were based on an improvement in progression-free survival without prolonging OS.

Bevacizumab is now considered an integral part of the chemotherapy schedule for several metastatic cancers.

On the other hand, accumulated evidence has suggested that regional deep capacitive hyperthermia (HT) can increase the cytotoxic effects of some anticancer agents by facilitating drug penetration into tissues and causing thermal destruction of cancer cells [14,15,16].

Potential synergic anti-angiogenic effects of HT are based on their ability to induce vasodilatation, improve oxygenation, and then reduce the hypoxic-inducible factor (HIF-1) molecule that is the main VEGF inducer. Significant improvement in clinical outcome has been demonstrated in randomized trials on HT alone or in addition to other treatment modalities for many cancer subtypes [15,16]. The synergic and adjuvant effect of HT was tested, for example, with gemcitabine for pancreatic cancer treatment, suggesting that the combination may improve the survival of advanced patients, without yielding any additional toxicity over those yielded by gemcitabine monotherapy [15].

Despite the interest in the potential role of HT to increase anti-tumor and anti-angiogenic effects, to the best of our knowledge, no data exist to date in literature which explore its combined use with bevacizumab.

The main aim of our study was to analyze the efficacy of anti-angiogenic-based chemotherapy associated to HT, in terms of objective response, in patients affected by metastatic colorectal, ovarian, and breast cancer. Secondary aims were to evaluate survival and treatment safety.

## 2. Methods

### 2.1. Patient Population

Between August 2014 and January 2017 a total of 23 patients with histologically proven colorectal (*n* = 16; adenocarcinoma), ovarian (*n* = 5; epithelial ovarian cancer), and breast (*n* = 2; invasive ductal carcinoma) cancer were enrolled in the present pilot-study.

All patients had progressive disease at the time of enrollment (stage III/IV), documented by Multidetector Contrast Enhancement Computed Tomography (MDCT) of the chest, abdomen, and pelvis and ^18^F-Fluorodeossiglucose-Positron Emission Tomography/CT (^18^F-FDG-PET/CT) scans, performed at baseline from the start of our therapy regimen. Most of them were previously subjected to at least one chemotherapy line (14/23, 60.9%), surgery (20/23, 87%), radiotherapy (5/23, 21.7%) and a combination of them in 18/23 patients (78.3%).

Eligibility of patients comprised criteria for bevacizumab-based chemotherapy included into the American Society of Clinical Oncology (ASCO) guidelines [17]. In particular, regarding colorectal cancer patients, V-KI-RAS2 Kirsten rat sarcoma viral oncogene homolog (KRAS) mutation status was considered to drive therapy: patients with a mutated status of KRAS were treated with folinic acid (leucovorin), 5-fluorouracil and oxaliplatin (FOLFOX) or folinic acid (leucovorin), 5-fluorouracil and irinotecan (FOLFIRI) schemes in combination with bevacizumab as up-front therapy. Conversely, patients with KRAS wild type status, already treated with Epidermal Growth Factor Receptor (EGFR) inhibitors plus FOLFOX or FOLFIRI, were considered suitable for the second-line bevacizumab-based chemotherapy.

For ovarian cancer, no tissue genetical markers were taken into account to select patients for therapy. Naïve patients for a first line of chemotherapy were enrolled and treated with the carboplatinum plus taxol plus bevacizumab combination.

As regard to breast cancer patients, HER-2 status was considered: only HER-2 negative patients on immunohistochemistry (score 1+) were enrolled for the first-line therapy including taxol plus bevacizumab combination. Patients with a borderline score on immunohistochemistry (score 2+) were further evaluated by fluorescent in situ hybridization (FISH) analysis. Those patients who showed amplification at FISH analysis were considered suitable for the treatment.

Further inclusion criteria included the following: an Eastern Cooperative Oncology Group (ECOG) performance status of 0–2, adequate bone marrow, renal, and hepatic function, and a lack of contraindications to hyperthermia treatment (deep venous/arterial thrombosis in the abdomen and/or pelvis).

Written informed consent was obtained from all patients before the start of therapy.

Patients’ characteristics are displayed in Table 1.

### 2.2. Bevacizumab, Antibody Characteristics

Bevacizumab (Avastin^®^) is a recombinant humanized IgG1 monoclonal antibody directed against VEGF, developed from a murine antihuman VEGF monoclonal antibody, and is 93% human and 7% murine. It belongs to the pharmacotherapeutic group of antineoplastic and immunomodulating agents.

Bevacizumab inhibits all the isoforms of VEGF-A, which is mainly responsible for pathologic angiogenesis and found to be up-regulated in various human tumors, preventing their binding to receptors and thereby inhibiting the VEGF/VEGF receptor signaling pathway [18].

VEGF inhibition has a number of effects on endothelial cells and the tumor vasculature. In the absence of VEGF, endothelial cells in immature vessels are unable to survive, causing regression of existing vessels [1]. Endothelial cells are also unable to grow and proliferate in the absence of VEGF, resulting in inhibition of new vessel formation. Some tumor blood vessels survive in the absence of VEGF, but anti-VEGF therapy makes them less permeable and morphologically more normal, thereby reducing the risk of tumor cells entering blood vessels and metastasizing. Normalization also reduces intratumoral pressure, which improves the penetration of macromolecules such as chemotherapy [19].

The half-life of bevacizumab has been estimated at approximately 20 days (range 10–50), after administration of doses of 1–20 mg/kg either weekly, every 2 weeks, or every 3 weeks. The pharmacokinetic properties of bevacizumab appear linear over the dose range 0.3–10 mg/kg. Time to reach steady-state is approximately 100 days and the accumulation ratio following a dose of 10 mg/kg every 2 weeks is 2.8. Bevacizumab clearance is higher in men than women (0.262 L/day vs. 0.207 L/day) and is higher in patients with greater tumor burden [20].

General toxicology studies in animal models have shown that the effects of bevacizumab on normal physiological processes are, as expected, based on its mechanism of action. Bevacizumab does not exacerbate common cytotoxic chemotherapy-associated side effects, such as myelosuppression, alopecia, diarrhea, nausea, and vomiting [21]. Clinically relevant side effects relating to bevacizumab therapy observed in patients with metastatic colorectal cancer (CRC) include hypertension, proteinuria, arterial thromboembolic events, wound healing complications, bleeding events, and gastrointestinal perforation [22].

Bevacizumab is approved by health authorities to be used in combination with chemotherapy in a number of cancer types. Detailed information on this medicinal product is available on the EMA website [23]. The posology and method of administration we used were in accordance with EMA guidelines and are described below.

### 2.3. Bevacizumab-Based Chemotherapy Regimen

All patients were treated with bevacizumab-based standard chemotherapy in relation to the histological cancer type (e.g., colorectal adenocarcinoma, epithelial ovarian cancer, and invasive ductal breast cancer) as the first or second line of chemotherapy for at least 2 cycles (range 2–24; mean 11.8), as showed in Table 2.

#### 2.3.1. Colorectal Cancer

Among 16 patients affected by metastatic colorectal cancer, 10/16 (62.5%) were subjected to a FOLFOX + Bevacizumab therapeutic scheme.

Oxaliplatin (85 mg/m^2^ dissolved in 250 mL sodium chloride 0.9%) was given on day 1 as an intravenous infusion over 60 min. Leucovorin (200 mg/m^2^ dissolved in 250 mL sodium chloride 0.9%) was intravenously applied over 60 min on day 1 and 2. 5-Fluorouracil was injected as an intravenous bolus at the dosage of 400 mg/m^2^ on day 1 and as an intravenous infusion over 22 h at the dosage of 600 mg/m^2^/die (dissolved in 250 mL sodium chloride 0.9%) on day 1 and 2.

Bevacizumab was associated to the previous scheme by administrating intravenously 5 mg/kg (dissolved in 250 mL sodium chloride 0.9%) over 90 (for the first time) and 60 (for the sequent) min on day 1, and repeated each 14 days.

Among the others metastatic colorectal cancer patients, 6/16 (37.5%) were subjected to FOLFIRI + Bevacizumab therapeutic scheme, that consisted of irinotecan (180 mg/m^2^ dissolved in 250 mL sodium chloride 0.9%) given on day 1 as intravenous infusion over 90 min, instead of oxaliplatin.

#### 2.3.2. Ovarian Cancer

In total, 5/23 (21.7%) patients affected by metastatic ovarian cancer were subjected to a Carboplatin + Taxol + Bevacizumab therapeutic scheme.

Carboplatin (AUC 5–7 dissolved in 250 mL sodium chloride 0.9%) was given on day 1 as intravenous infusion over 60 min. In addition, paclitaxel (175 mg/m^2^ dissolved in 250 mL sodium chloride 0.9%) was intravenously applied over 3 h on day 1. This scheme was repeated each 21 days.

Bevacizumab was associated to the previous scheme by administrating intravenously 15 mg/kg (dissolved in 250 mL sodium chloride 0.9%) over 90 (for the first time) and 60 (for the sequent) min on day 1, and repeated each 21 days.

#### 2.3.3. Breast Cancer

In total, 2/23 (8.7%) patients affected by metastatic breast cancer were subjected to a Taxol + Bevacizumab therapeutic scheme.

Paclitaxel (90 mg/m^2^ dissolved in 250 mL sodium chloride 0.9%) was intravenously applied over 60 min on day 1, 8, 15, and 28 and so on.

Bevacizumab was associated to the previous scheme by administrating it intravenously at a dosage of 10 mg/kg (dissolved in 250 mL sodium chloride 0.9%) over 90 (for the first time) and 60 (for the sequent) min on day 1, and repeated every 2 weeks.

As anti-emetic prophylaxis patients received a serotonin-5HT3-antagonist, hematological and non-hematological toxicity was assessed according to Common Terminology Criteria for Adverse Events (CTCAE) version 4.0, and recorded at each cycle of treatment.

Treatment was postponed for 1 week or more if white blood cell counts were below 2 × 10^3^ µL, granulocytes were below 0.5 × 10^3^ µL, and platelets were below 100 × 10^3^ µL. Chemotherapy regimen was reduced in the following cycle to 75% if nadir of granulocytes was <1.5 × 10^3^ µL, platelets <100 × 10^3^ µL, or any non-hematological toxicity grade 3 occurred.

Treatment was continued until progressive disease or unacceptable drug-related toxicities.

### 2.4. Regional Deep Capacitive Hyperthermia (HT)

HT was performed according to the European Society of Hyperthermic Oncology (ESHO) guidelines for quality and safety assurance [24], by using the Oncotherm EHY-2000 medical device (Oncotherm GmbH, Traisdorf, Germany).

HT was performed with capacitive electrodes at 80–110 W for 50 min as therapeutic time. A large, water-cooled bolus asymmetric electrode (30 cm in diameter) was used.

All patients received HT treatment once a week, when possible in combination with bevacizumab administration, for at least 4 sessions (range 4–32; mean 21.9), as reported in Table 2.

The target area of HT was abdomen (*n* = 15) for liver, spleen, abdominal lymph nodes, adrenal gland, or peritoneal carcinosis as sites of metastasis; pelvis (*n* = 1) for site of disease in ovary; thorax (*n* = 7) for site of metastasis in lung. If more than one target area was present, a maximum of two target points were used alternately for each cycle (*n* = 6).

Patients were carefully instructed to report any discomfort during treatment. Moreover, late HT-associated adverse events were recorded for each patient. HT treatment was stopped if an adverse event occurred or if desired by patients.

### 2.5. Treatment Response Evaluation

**Figure 1:**
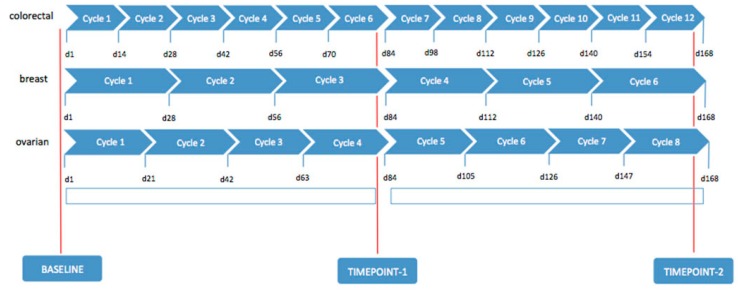
Treatment schedule and evaluation timing for colorectal, breast, and ovarian cancer patients respectively.

A clinical-instrumental evaluation, based on general condition, clinical signs, laboratory tests, including tumor biological circulation markers (Ca19.9 and Carcino-Embryonic Antigen (CEA) for colorectal cancer, Ca15.3 and CEA for breast cancer, Ca125 for ovarian cancer), ultrasound, chest, abdomen, and pelvis MDCT scan, were required before the start of treatment and at 80 days (timepoint-1) and at 160 days (timepoint-2) from starting therapy, in order to assess tumor response, monitor safety, for compliance, and to determine side effects (Figure 1).

In addition, all patients performed ^18^F-FDG PET/CT at baseline and at timepoint-2.

All MDCT and ^18^F-FDG PET/CT exams were evaluated by two independent radiologists and nuclear physicians.

Dimensional tumor measurements and the evaluation of the enhancement pattern of the target lesion were performed on MDTC at baseline and repeated at timepoint-1 and timepoint-2, assessing tumor response rates according to modified Response Evaluation Criteria for Solid Tumors (mRECIST-Version 1.1) [25].

Treatment response was categorized as Complete Response (CR), Partial Response (PR), Stable Disease (SD) and Progressive Disease (PD).

Disease response rate (DRR) was considered as the proportion of patients who had the best response rating of CR, PR, or SD, according to mRECIST.

Patients were strictly followed after timepoint-2 evaluation by clinical and laboratory examination each month and by performing MDCT every 3 months.

OS was specified as the time from the start of treatment until the date of death. Time to Progression (TTP) was defined as the time from the start of treatment until progression of disease or death.

### 2.6. Statistical Analysis

At timepoint-1 and timepoint-2, patients in CR, PR, and SD were classified as responders (R), while patients in PD were non responders (NR).

Fisher’s exact test was used to assess the association between response groups (R/NR) and histological cancer type (colorectal adenocarcinoma vs. epithelial ovarian cancer plus invasive ductal breast cancer), ECOG pre-treatment (0 vs. 1), ECOG post-treatment (0 vs. 1), and chemotherapy-related and/or hyperthermia-related adverse events (presence vs. absence).

Variation of tumor biological circulation markers from baseline to timepoint-2 between response groups (R/NR) was evaluated by *U* Mann-Whitney test.

Chi-squared for linear trend test was performed to evaluated the association between response groups (R/NR) and the number of previous treatment (none, 1, 2, 3), number of chemotherapy cycles (<6, 6, 12, >12), number of hyperthermia sessions (<12, 12, 24, >24), and lines of chemotherapy (I, II).

Finally, survival curves were estimated by the Kaplan-Meier method, with differences assessed by the log-rank test. Significant difference was defined as *p* < 0.05.

All statistical analyses were performed using SPSS Statistics software (Version 23.0, Armonk, NY 10504-1722, USA).

## 3. Results

### 3.1. Patient Characteristics

The majority of patients showed metastasized disease stage IV (*n* = 22, see Table 1); the one patient at stage III was affected by ovarian cancer.

Despite this highly palliative patient population, most of patients presented an ECOG performance status of 0 (*n* = 19, see Table 1) and only 4 had an ECOG performance status of 1.

A total of 255 chemotherapy cycles with a mean of 11 cycles per patient (range 2–16) and a total of 490 hyperthermia sessions with a mean of 21 sessions per patient (range 4–32) were given.

Eighteen patients (78.3%) completed all cycles of chemo-HT therapy at timepoint-2; four of them, who achieved SD, further prolonged the therapeutic scheme as maintenance therapy until imaging evaluation.

Five patients received less cycles of chemo-HT therapy because of disease progression during treatment; two of them did not complete timepoint-1 evaluation due to non-treatment-related death.

The mean values of biological circulating tumor markers collected at baseline, timepoint-1, and timepoint-2 are showed in Table 3.

The mean follow-up period, until last observation, for those patients who completed timepoint-2 evaluation was 98 days (range: 29–411).

### 3.2. Toxicity

Bevacizumab-based chemotherapy schemes were generally well tolerated: only one patient needed to stop the FOLFOX schedule for adverse events and continued treatment with oxaliplatin and bevacizumab alone. Nonetheless, he achieved a good response to treatment.

Side effects were limited to anemia (2 cases), leucopenia (3 cases), nausea and vomiting, which was the leading non-hematological side effects occurring in a total of 10 patients, asthenia (4 cases), peripheral sensory neuropathy (3 cases), high blood pressure (1 case), epistaxis (1 case) and gastrointestinal discomfort (2 cases). However, none of the listed side effects led to a break of treatment.

The addition of HT did not result in additional adverse effects on chemotherapy-related toxicity. Mild position-related pain during HT sessions was the leading side effect referred to by seven patients. Power-related pain occurred in two cases during the first session and dissolved by power adjustment.

One patient stopped hyperthermia ahead of time as he/she developed abdominal pain, arterial thrombosis, and ascites, which represents relative exclusion criteria for hyperthermia treatment.

No patient required a significant treatment interruption because of treatment complication and there was no treatment-related death.

### 3.3. Treatment Response

Two of the twenty-three patients died due to progressive disease before completing the timepoint-1 evaluation.

Therefore, clinical-instrumental evaluation at timepoint-1 was evaluable in 21/23 (91.3%) patients: PR was detected in 7/21 (33.3%) patients, SD in 11/21 (52.4%) patients, and PD in 3/21 (14.3%) patients, with a DRR of 85.7% (Table 4).

**Figure 2:**
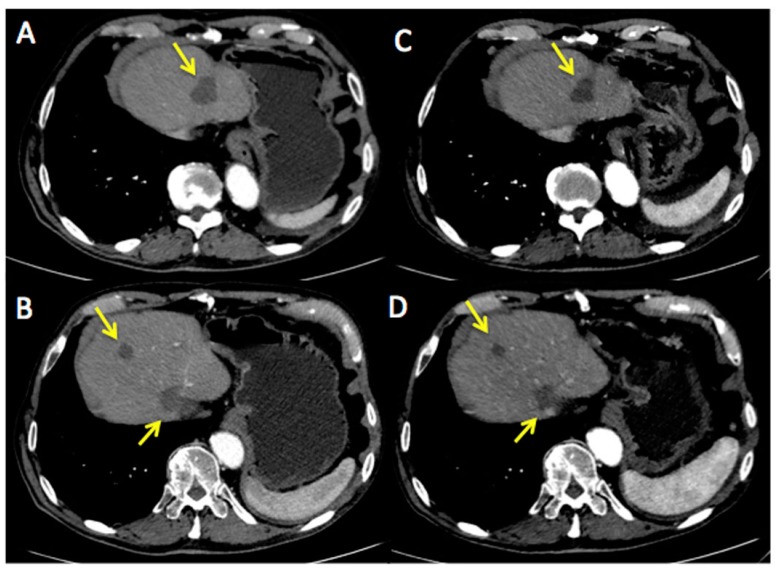
A 71-year-old male affected by colorectal cancer with liver metastasis, subjected to 6 cycles of Bevacizumab-based chemotherapy and 10 hyperthermia sessions on the abdomen as second-line. Baseline Multidetector Contrast Enhancement Computed Tomography (MDCT) (**A**,**B**) showed metastasis in the II and VIII segment of the liver and caudate lobe (maximum diameter: 47 mm, yellow arrows). Timepoint-1 MDCT (**C**,**D**) evaluation demonstrated stable size of liver metastasis (maximum diameter: 44 mm, yellow arrows). According to mRECIST, patient was classified as SD. Ca19.9 and Carcino-Embryonic Antigen (CEA) values evaluated at baseline were 99 UI/mL and 65 ng/mL, respectively; while Ca19.9 and CEA values evaluated at timepoint-1 were 81 UI/mL and 60 ng/mL, respectively. Side effects reported were limited to nausea and mild position-related pain during HT sessions.

Figure 2 represents an exemplar case of a patient judged as SD at the timepoint-1 evaluation.

The three patients who were considered in PD at timepoint-1 did not continue the treatment: one patient changed treatment scheme, while the other two died for complications soon after.

Eighteen out of twenty-one (85.7%) patients completed the clinical-instrumental evaluation at timepoint-2: CR was achieved in 6/18 (33.3%) patients, PR in 2/18 (11.1%) patients, SD in 5/18 (27.8%) patients, and PD in 5/18 (27.8%) patients, with a DRR of 72.2% (Table 4).

DRR decreased in 13.5% from timepoint-1 to timepoint-2 treatment response evaluations.

**Figure 3:**
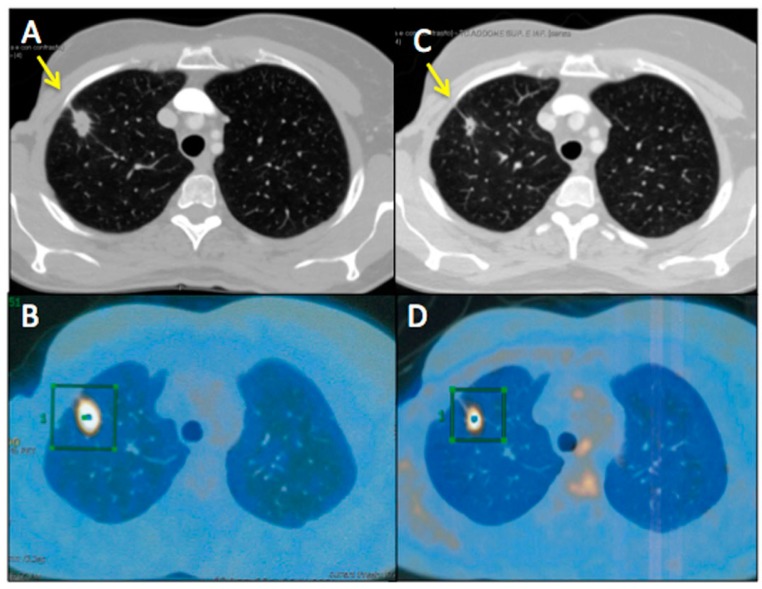
A 55-year-old female affected by breast cancer with lung metastasis, subjected to 12 cycles of Bevacizumab-based chemotherapy and 24 hyperthermia sessions on the thorax, as first-line. Baseline Multidetector Contrast Enhancement Computed Tomography (MDCT) (**A**) showed metastasis in the superior right lobe of the lung (diameter: 17 mm × 12 mm, yellow arrow), confirmed by the increased ^18^F-Fluorodeossiglucose (^18^F-FDG) uptake on Positron Emission Tomography/CT (PET/CT) images in the same sites (green square, **B**). Timepoint-2 MDCT (**C**) evaluation demonstrated size decrease of lung metastasis (diameter 14 mm × 7 mm, yellow arrow) with ^18^F-FDG uptake decrease on PET/CT images (green square, **D**). According to mRECIST, patient was classified as PR. Ca15.3 and CEA values evaluated at baseline were 830 UI/mL and 135 ng/mL, respectively; while Ca15.3 and CEA values evaluated at timepoint-2 were 115 UI/mL and 40 ng/mL, respectively. Side effects reported were limited to asthenia and peripheral sensory neuropathy.

**Figure 4:**
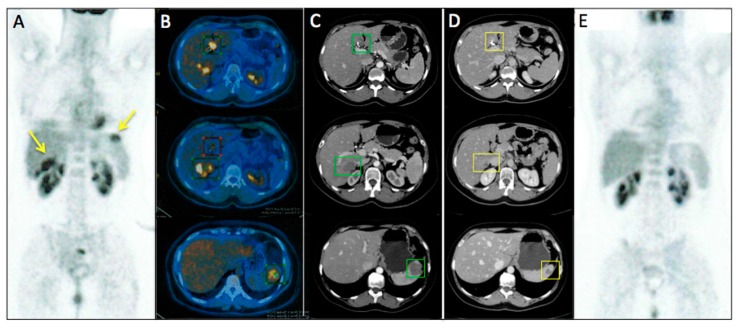
A56-year-old female affected by ovarian cancer with liver and spleen metastasis, already treated with 12 cycles of Bevacizumab-based chemotherapy and 24 hyperthermia sessions on the abdomen, as first-line. Baseline MDCT (**C**) showed metastasis in III and IV segment of the liver (maximum diameter: 77 mm, green squares) and in the upper spleen (maximum diameter: 61 mm, green square). Baseline whole body ^18^F-FDG PET/CT (**A**) confirmed liver and spleen involvement by the increased ^18^F-FDG uptake (yellow arrows) detectable also on axial fused PET/CT images (**B**) in the same sites (green and red squares). Timepoint-2 MDCT (**D**) evaluation demonstrated significant size decrease of liver and spleen metastasis (yellow squares) with no evidence of ^18^F-FDG uptake on whole body PET/CT (**E**). According to mRECIST, patient was classified as CR. Ca125 value evaluated at baseline was 790 UI/mL; while Ca125 value evaluated at timepoint-2 was 12 UI/mL. Side effects reported were limited to asthenia and high blood pressure.

Figure 3 and Figure 4 represent two exemplar cases of patients judged to be PR and CR, respectively, at timepoint-2 evaluation.

Fisher’s exact test showed that response groups (R/NR) were related neither to histological cancer type (*p* = 0.077) nor to ECOG pre-treatment (*p* = 0.596), to ECOG post-treatment (*p* = 0.057), nor to adverse chemotherapy-related (*p* = 0.057) or HT-related (*p* = 0.092) events.

Among tumor biological circulation markers collected, only CEA and Ca19.9 were considered suitable for the statistical analysis because of their higher number. U Mann-Whitney test showed that the variation from baseline to timepoint-2 between response groups (R/NR) was statistically significant, both for CEA (*p* = 0.001) and Ca19.9 (*p* = 0.004).

Any association between response groups (R/NR) and number of previous treatment (X^2^ = 5.596, *p* = 0.133) existed.

Conversely, chi-squared for linear trend test demonstrated that the percentage of responders grew in relation to the number of chemotherapy cycles (X^2^_lin_ = 5.875, *p* = 0.015) and to number of HT sessions (X^2^_lin_ = 10.188, *p* < 0.001) performed. In addition, the percentage of responders was inversely associated with the lines of chemotherapy (X^2^_lin_ = 4.379, *p* = 0.036).

**Figure 5:**
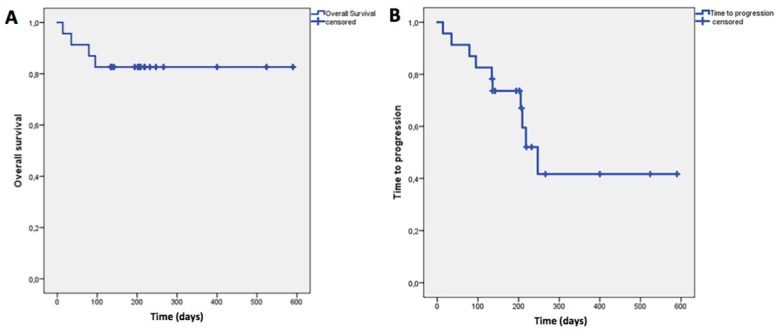
(**A**) Overall Survival (OS), defined as the time from the start of treatment until the date of death; (**B**) Time to Progression (TTP), assessed as the time from the start of treatment until progression of disease or death. The mean OS was 497 days (95% CI: 414–580 ± 42.3) while the mean TTP was 339 days (95% CI: 229–449 ± 56).

The mean OS was 497 days (95%CI: 414–580 ± 42.3) while the mean TTP was 339 days (95% CI: 229–449 ± 56), as showed in Figure 5.

**Figure 6:**
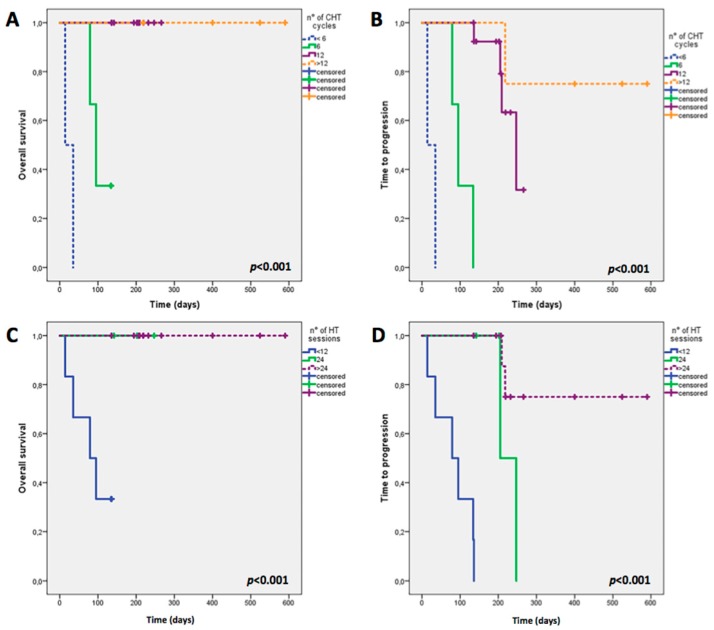
Overall Survival (OS) and Time to Progression (TTP) in relation to number of chemotherapy cycles (<6, 6, 12, >12) (**A**,**B**) and number of hyperthermia sessions (<12, 12, 24, >24) (**C**,**D**). CHT, chemotherapy; HT, hyperthermia. Both OS and TTP were influenced by the number of chemotherapy cycles (Log Rank = 35.406, *p* < 0.001; Log Rank = 43.995, *p* < 0.001) and HT sessions performed (Log Rank = 15.470, *p* < 0.001; Log Rank = 32.479, *p* < 0.001) (**A**–**D**).

Both OS and TTP were influenced by the number of chemotherapy cycles (Log Rank = 35.406, *p* < 0.001; Log Rank = 43.995, *p* < 0.001) and HT sessions performed (Log Rank = 15.470, *p* < 0.001; Log Rank = 32.479, *p* < 0.001) as showed in Figure 6 A–D; on the contrary, neither lines of chemotherapy (Log Rank = 3.315, *p* = 0.191; Log Rank = 4.198, *p* = 0.123) nor histological cancer type (Log Rank = 1.941, *p* = 0.164; Log Rank = 2.651, *p* = 0.104) influenced OS and TTP significantly.

## 4. Discussion

Literature reports that the addition of bevacizumab to standard chemotherapy regimens improves survival duration for patients with previously treated metastatic colorectal cancer [5,6,26,27,28].

As reported by Giantonio B.J. et al. [27], metastatic colorectal cancer treated with bevacizumab in combination with FOLFOX4 had a median survival of 12.9 months compared with 10.8 months for those treated with FOLFOX4 alone (hazard ratio = 0.75; *p* < 0.0011). In addition, the combination of bevacizumab and FOLFOX4 resulted in a statistically significant improvement in progression-free survival (PFS) compared with those treated with chemotherapy alone (7.3 vs. 4.7 months; hazard ratio for progression = 0.61; *p* < 0.0001). Improvements in clinical efficacy have also been described when bevacizumab is added to a standard chemotherapy regimen for several other metastatic cancers.

According to the AVADO trial, bevacizumab improved efficacy, including one-year OS rates (71% vs. 65%) in metastatic breast cancer. The PFS was 8.1 months with bevacizumab vs. 5.4 months with chemotherapy alone [4,29].

The GOG-218 trial, which enrolled 1873 patients with FIGO stage III–IV ovarian cancer with macroscopic residual disease after primary surgery, showed a significant improvement in PFS with the addition of bevacizumab [30,31]. Median PFS, which was the primary endpoint of the trial, was 10.3 months in the control group, 11.2 months in the bevacizumab-initiation group, and 14.1 months in the bevacizumab-throughout group. The hazard of progression or death was significantly lower in the bevacizumab-throughout group compared with that of the control group (hazard ratio = 0.72; *p* < 0.001) [32].

As an anti-angiogenic agent, bevacizumab interferes with the “angiogenic switch”, the crucial step in neo-vascularization, that allows tumors to usurp the growth mechanisms of normal vascular endothelial cells and develop to macroscopic size [33,34]. This process is mediated by the interaction of VEGFs with their membrane-bound receptors (VEGF-Rs). Therapeutic disruption of tumor neo-vascularization can be achieved by using bevacizumab, which recognizes human VEGF, thereby eliminating the ligands required for VEGF-R activation and the mitogenic and permeability-enhancing stimuli necessary for neo-vascularization. Given that bevacizumab is a monoclonal antibody, it is distributed to highly perfused areas with a linear kinetic profile [11,35]. The increased microvascular permeability induced by HT, that facilitates the bevacizumab distribution in cancer tissues emphasizing its therapeutic efficacy, represents just one of the potential synergic antiangiogenic and proapoptotic effects for combining bevacizumab-based chemotherapy and HT.

In fact, HT itself is able to inhibit angiogenesis through the direct endothelial cell damage caused by the absorption of electric field energy in the extracellular liquid, with a subsequent temperature gradient between the extra- and intracellular compartments, which destroys cancer cell membranes [36]. The denaturation of membrane proteins alters the permeability of tumor cells and permits the easier entrance of chemotherapeutic agents, which have already arrived in greater tumor quantity due to HT-induced vasodilation, damaging them irreversibly and leading to necrosis or apoptosis.

As malignant cells typically exhibit relatively rigid membranes, due to increased phospholipid concentration, and the conductivity as well as the dielectric constant of the extracellular matrix in malignant tissue are higher than in the normal tissue, this technique results in selective tumor tissue destruction [16,37].

On the other hand, HT causes vasodilatation, reduces hypoxia, and then reduces the hypoxic-inducible factor (HIF-1) molecule, which is the main VEGF inducer, promoting the oxygen supply and increasing the local immune response. In particular, detritus derived from denaturation of membrane proteins or nucleid acids represent antigens, which stimulate the anti-tumoral response [37].

On these premises is based the rationale for evaluating the combined use of bevacizumab-based chemotherapy and HT in the present study, which has not yet been explored in literature to the best of our knowledge.

The main aim of our study was to analyze the efficacy of anti-angiogenic-based chemotherapy associated to local deep capacitive hyperthermia of enrolled patients, in terms of objective response. In this regard, our pilot-study showed that the combination demonstrated noteworthy antitumor activity with almost 28% of patients achieving SD, 11% PR, and 33% achieving CR. The overall DRR at timepoint-2 exceeded 72%, which resembles that reported in literature [38], even if it is not comparable due to the miscellaneous histological types in our sample. Our analysis demonstrated that a better response was directly associated with a higher number of chemotherapy cycles (*p* = 0.015) and number of HT sessions (*p* < 0.001) performed.

The statistical significance demonstrated for CEA (*p* = 0.001) and Ca19.9 (*p* = 0.004) in our sample suggested that these biological circulating tumor biomarkers could be associated together with imaging modalities in the treatment response assessment, particularly for a closer monitoring.

An interesting finding from our results is the significant association between response groups (R/NR) and lines of chemotherapy found (*p* = 0.036); response to therapy in patients who performed bevacizumab-based chemotherapy plus HT as first-line therapy were significantly better than in those who perform it as a second-line. In fact, most patients who performed treatment as a second-line, failed treatment for PD; they were all patients affected by colorectal cancer. Conversely, most patients who performed treatment as first-line therapy achieved CR or PR; they were patients affected by breast and especially ovarian cancer. This result is in line with previous studies that explored the addition of bevacizumab to front-line chemotherapy, concluding that it is feasible and well tolerated, encouraging its employment [22,29,31].

Improvements of survival, while maintaining safety of the treatment, represented secondary aims of this study. Our results showed an interesting OS mean value of 497 days (16.6 months) and TTP mean value of 339 days (11.3 months). To this regard, it was not possible to calculate the median values of OS due to the small number of deaths observed in our sample. For this reason, it is not reasonable to compare our results with the OS and TTP median values mainly reported in the literature. As a consequence, this preliminary data has to be considered with caution.

On the other hand, despite the limitations of our study that consisted of small numbers and a heterogeneous sample, it is important to remark that our results represent the first clinical data on this topic and suggest the potential benefit of HT plus bevacizumab-based chemotherapy combination.

The main result of our study concerned the positive association we found between the percentage of responders and the number of chemotherapy cycles (*p* = 0.015) and HT sessions (*p* < 0.001) performed, and the significantly influence of OS (*p* < 0.001) and TTP (*p* < 0.001), as showed in Figure 6.

Those patients who performed more cycles of chemotherapy as well as and more sessions of HT showed a significantly better OS and TTP compared to those who did not.

These results confirm the efficacy of bevacizumab-based chemotherapy and speculatively suggest that, thanks to the well-known additive effects, HT may enhance the treatment, which could have an impact on survival.

Moreover, an important advantage of the combination of bevacizumab-based chemotherapy and HT is the minimal onset of side effects [16]. HT was well tolerated in our sample without additional adverse effects on chemotherapy-related toxicity. The good tolerability of HT is an important goal, especially in heavily pre-treated patients, that makes treatment safe and repeatable.

## 5. Conclusions

This pilot study confirmed that bevacizumab-based chemotherapy is an important therapeutic option in different metastatic cancer patients. Although further larger studies are needed to confirm the data, our preliminary results suggest that the addition of regional deep capacitive hyperthermia is feasible and well tolerated and could enhance the treatment, improving tumor response without additional adverse effects.

## Figures and Tables

**Table 1:** Patients’ clinical and pathological characteristics. ECOG, Eastern Cooperative Oncology Group; CHT, chemotherapy.

Characteristics	Enrolled Patients (*n* = 23)	%
Gender		
Male	7	30.4%
Female	16	69.6%
Median age, years	60.2	Range 42–79
ECOG performance status		
0	19	82.6%
1	4	17.4%
Primitive cancer		
Colorectal	16	69.6%
Breast	2	8.7%
Ovarian	5	21.7%
Stage at study entry		
III	1	4.3%
IV	22	95.7%
Site of metastases		
Liver	13	56.5%
Lung	7	30.4%
Lymph nodes	8	34.8%
Peritoneal Carcinosis	4	17.4%
Bone	5	21.7%
Ovary	1	4.3%
Adrenal gland	1	4.3%
Spleen	1	4.3%
No. of involved site for pts		
1	12	52.2%
2	6	26.1%
3	5	21.7%
Previous CHT lines		
yes	14	60.9%
no	9	39.1%

**Table 2:** Patients’ treatment characteristics. CHT, chemotherapy; FOLFOX, folinic acid (leucovorin), 5-fluorouracil, oxaliplatin; FOLFIRI, folinic acid (leucovorin), 5-fluorouracil, irinotecan; HT, hyperthermia.

Characteristics	Patients (*n* = 23)
**Treatment scheme/CHT lines**	
Colorectal	
FOLFOX + Bevacizumab	10
FOLFIRI + Bevacizumab	6
Breast	
Taxol + Bevacizumab	2
Ovarian	
Carboplatin + Taxol + Bevacizumab	5
**CHT lines**	
I	9
II	14
**No. of cycles of CHT**	
<6	2
6	3
12	14
>12	4
**No. of sessions of HT**	
<12	6
12	0
24	7
>24	10

**Table 3:** Biological circulating tumor markers collected at baseline, timepoint-1, and timepoint-2.

Tumor Markers	Baseline (*n* = 23)		Timepoint-1 (*n* = 21)		Timepoint-2 (*n* = 18)	
	Mean Value	Range	Mean Value	Range	Mean Value	Range
Ca19.9 (UI/mL)	659.9	(18–1730)	643.23	(22–2500)	505.54	(20–1800)
CEA (ng/mL)	449.83	(5–3200)	459.56	(7–3250)	545.85	(6–4100)
Ca15.3 (UI/mL)	865	(830–900)	320	(120–520)	49.5	(45–54)
Ca125 (UI/mL)	1981.6	(790–4138)	750.6	(50–2113)	35	(12–50)

**Table 4:** Clinical response assessment according to the modified Response Evaluation Criteria for Solid Tumors (mRECIST) at timepoint-1 and timepoint-2. CR, Complete Response; PR, Partial Response; SD, Stable Disease; PD, Progressive Disease; DRR, Disease Response Rate = CR + PR + SD.

Clinical Response	Timepoint-1 (*n* = 21)	%	Timepoint-2 (*n* = 18)	%
CR	/	/	6	33.3
PR	7	33.3	2	11.1
SD	11	52.4	5	27.8
PD	3	14.3	5	27.8
DRR	18	85.7	13	72.2

## References

[1] Ferrara N., Gerber H.P., LeCouter J. (2003). The biology of VEGF and its receptors. Nat. Med..

[2] Dvorak H.F. (2002). Vascular permeability factor/vascular endothelial growth factor: A critical cytokine in tumor angiogenesis and a potential target for diagnosis and therapy. J. Clin. Oncol..

[3] Saponaro C., Malfettone A., Ranieri G., Danza K., Simone G., Paradiso A., Mangia A. (2013). VEGF, HIF-1α Expression and MVD as an Angiogenic Network in Familial Breast Cancer. PLoS ONE.

[4] Kristensen T.B., Knutsson M.L., Wehland M., Laursen B.E., Grimm D., Warnke E., Magnusson N.E. (2014). Anti-Vascular Endothelial Growth Factor Therapy in Breast Cancer. Int. J. Mol. Sci..

[5] Hurwitz H., Fehrenbacher L., Novotny W., Cartwright T., Hainsworth J., Heim W., Berlin J., Baron A., Griffing S., Holmgren E. (2004). Bevacizumab plus irinotecan, fluorouracil, and leucovorin for metastatic colorectal cancer. N. Engl. J. Med..

[6] Saif M.W., Merritt J., Robbins J., Stewart J., Schupp J. (2006). Phase III multicenter randomized clinical trial to evaluate the safety and efficacy of CoFactor/5-fluorouracil/bevacizumab versus leucovorin/5-fluorouracil/bevacizumab as initial treatment for metastatic colorectal carcinoma. Clin. Colorectal Cancer.

[7] Johnson D.H., Fehrenbacher L., Novotny W.F., Herbst R.S., Nemunaitis J.J., Jablons D.M., Langer C.J., DeVore R.F., Gaudreault J., Damico L.A. (2004). Randomized phase II trial comparing bevacizumab plus carboplatin and paclitaxel with carboplatin and paclitaxel alone in previously untreated locally advanced or metastatic non-small-cell lung cancer. J. Clin. Oncol..

[8] Tyagi P. (2005). Bevacizumab, when added to paclitaxel/carboplatin, prolongs survival in previously untreated patients with advanced non-small-cell lung cancer: Preliminary results from the ECOG 4599 trial. Clin. Lung Cancer.

[9] Monk B.J., Choi D.C., Pugmire G., Burger R.A. (2005). Activity of bevacizumab (rhuMAB VEGF) in advanced refractory epithelial ovarian cancer. Gynecol. Oncol..

[10] Aghajanian C. (2006). The role of bevacizumab in ovarian cancer—An evolving story. Gynecol. Oncol..

[11] Pories S.E., Wulf G.M. (2010). Evidence for the role of bevacizumab in the treatment of advanced metastatic breast cancer: A review. Breast Cancer.

[12] Wehland M., Bauer J., Infanger M., Grimm D. (2012). Target-based anti-angiogenic therapy in breast cancer. Curr. Pharm. Des..

[13] Miller K.D., Chap L.I., Holmes F.A., Cobleigh M.A., Marcom P.K., Fehrenbacher L., Dickler M., Overmoyer B.A., Reimann J.D., Sing A.P. (2005). Randomized phase III trial of capecitabine compared with bevacizumab plus capecitabine in patients with previously treated metastatic breast cancer. J. Clin. Oncol..

[14] Engelhardt R. (1987). Hyperthermia and drugs. Recent Results Cancer Res..

[15] Ishikawa T., Kokura S., Sakamoto N., Ando T., Imamoto E., Hattori T., Oyamada H., Yoshinami N., Sakamoto M., Kitagawa K. (2012). Phase II trial of combined regional hyperthermia and gemcitabine for locally advanced or metastatic pancreatic cancer. Int. J. Hyperth..

[16] Gadaleta-Caldarola G., Infusino S., Galise I., Ranieri G., Vinciarelli G., Fazio V., Divella R., Daniele A., Filippelli G., Gadaleta C.D. (2014). Sorafenib and locoregional deep electro-hyperthermia in advanced hepatocellular carcinoma: A phase II study. Oncol. Lett..

[17] American Society of Clinical Oncology Recommendations on Adjuvant Chemotherapy for Stage II Colon Cancer. http://ascopubs.org/doi/abs/10.1200/JCO.2004.05.063.

[18] Ferrara N., Hillan K.J., Gerber H.P., Novotny W. (2004). Discovery and development of bevacizumab, an anti-VEGF antibody for treating cancer. Nat. Rev. Drug Discov..

[19] Lee T.H., Avraham H.K., Jiang S., Avraham S. (2003). Vascular endothelial growth factor modulates the transendothelial migration of MDA-MB-231 breast cancer cells through regulation of brain microvascular endothelial cell permeability. J. Biol. Chem..

[20] Krämer I., Lipp H.P. (2007). Bevacizumab, a humanized anti-angiogenic monoclonal antibody for the treatment of colorectal cancer. J. Clin. Pharm. Ther..

[21] Gordon M.S., Margolin K., Talpaz M., Sledge G.W., Holmgren E., Benjamin R., Stalter S., Shak S., Adelman D. (2001). Phase I safety and pharmacokinetic study of recombinant human anti-vascular endothelial growth factor in patients with advanced cancer. J. Clin. Oncol..

[22] Kabbinavar F.F., Schulz J., McCleod M., Patel T., Hamm J.T., Hecht J.R., Mass R., Perrou B., Nelson B., Novotny W.F. (2005). Addition of bevacizumab to bolus fluorouracil and leucovorin in first-line metastatic colorectal cancer: Results of a randomized phase II trial. J. Clin. Oncol..

[23] Annex I. Summary of Product Characteristics. http://www.ema.europa.eu/docs/en_GB/document_library/EPAR_-_Product_Information/human/000582/WC500029271.pdf.

[24] Bruggmoser G., Bauchowitz S., Canters R., Crezee H., Ehmann M., Gellermann J., Lamprecht U., Lomax N., Messmer M.B., Ott O. (2011). ESHO Technical Committee in the Interdisciplinary Working Group Hyperthermia (IAH) in the German Cancer Society: Quality assurance for clinical studies in regional deep hyperthermia. Strahlenther. Onkol..

[25] Eisenhauer E.A., Therasse P., Bogaerts J., Schwartz L.H., Sargent D., Ford R., Dancey J., Arbuck S., Gwyther S., Mooney M. (2009). New response evaluation criteria in solid tumours: Revised RECIST guideline (version 1.1). Eur. J. Cancer..

[26] Marech I., Ammendola M., Gadaleta C., Zizzo N., Oakley C., Gadaleta C.D., Ranieri G. (2014). Possible biological and translational significance of mast cells density in colorectal cancer. World J. Gastroenterol..

[27] Giantonio B.J., Catalano P.J., Meropol N.J., O’Dwyer P.J., Mitchell E.P., Alberts S.R., Schwartz M.A., Benson A.B., Eastern Cooperative Oncology Group Study E3200 (2007). Bevacizumab in combination with oxaliplatin, fluorouracil, and leucovorin (FOLFOX4) for previously treated metastatic colorectal cancer: Results from the Eastern Cooperative Oncology Group Study E3200. J. Clin. Oncol..

[28] Kabbinavar F., Hurwitz H.I., Fehrenbacher L., Meropol N.J., Novotny W.F., Lieberman G., Griffing S., Bergsland E. (2003). Phase II, randomized trial comparing bevaci- zumab plus fluorouracil (FU)/leucovorin (LV) with FU/LV alone in patients with metastatic colorectal cancer. J. Clin. Oncol..

[29] Miles D.W., Diéras V., Cortés J., Meropol N.J., Novotny W.F., Lieberman G., Griffing S., Bergsland E. (2013). First-line bevacizumab in combination with chemotherapy for her2-negative metastatic breast cancer: Pooled and subgroup analyses of data from 2447 patients. Ann. Oncol..

[30] Oza A.M., Cook A.D., Pfisterer J., Embleton A., Ledermann J.A., Pujade-Lauraine E., Kristensen G., Carey M.S., Beale P., Cervantes A. (2015). Standard chemotherapy with or without bevacizumab for women with newly diagnosed ovarian cancer (ICON7): Overall survival results of a phase 3 randomised trial. Lancet Oncol..

[31] Burger R.A., Brady M.F., Bookman M.A., Fleming G.F., Monk B.J., Huang H., Mannel R.S., Homesley H.D., Fowler J., Greer B.E. (2011). Incorporation of bevacizumab in the primary treatment of ovarian cancer. N. Engl. J. Med..

[32] Garcia A., Singh H. (2013). Bevacizumab and ovarian cancer. Ther. Adv. Med. Oncol..

[33] Hanahan D., Folkman J. (1996). Patterns and emerging mechanisms of the angiogenic switch during tumorigenesis. Cell.

[34] Ribatti D., Ranieri G., Basile A., Azzariti A., Paradiso A., Vacca A. (2012). Tumor endothelial markers as a target in cancer. Expert Opin. Ther. Targets.

[35] Herbst R.S. (2006). Therapeutic options to target angiogenesis in human malignancies. Expert Opin. Emerg. Drugs.

[36] Roca C., Primo L., Valdembri D., Cividalli A., Declerck P., Carmeliet P., Gabriele P., Bussolino F. (2003). Hyperthermia inhibits angiogenesis by a plasminogen activator inhibitor 1-dependent mechanism. Cancer Res..

[37] Fiorentini G., Szasz A. (2006). Hyperthermia today: Electric energy, a new opportunity in cancer treatment. J. Cancer Res. Ther..

[38] Micha J., Goldstein B., Rettenmaier M., Genesen M., Graham C., Bader K., Lopez K.L., Nickle M., Brown J.V. (2007). A phase II study of outpatient first-line paclitaxel, carboplatin, and bevacizumab for advanced-stage epithelial ovarian, peritoneal, and fallopian tube cancer. Int. J. Gynecol. Cancer..

